# Focus on Tumours in Pet Animals

**DOI:** 10.3390/vetsci12030266

**Published:** 2025-03-12

**Authors:** Louise van der Weyden

**Affiliations:** Sanger Institute, Wellcome Genome Campus, Cambridge CB10 1SA, UK; lvdw@sanger.ac.uk

Pet animals, in particular dogs and cats, are often regarded as family members, and, as such, most owners want the best care possible for their companions. Sadly, statistics show that one in four dogs and one in five cats will develop cancer during their lifetime, and unfortunately, in many cases, there are limited treatment options available. Thus, more research is needed to investigate and characterise the range of tumours that can develop in companion animals, as it is only through a thorough understanding of the biology and underlying genetics of these tumours that we can hope to develop better diagnostic tools, prognostic markers, and therapeutic strategies. To this end, the Veterinary Sciences Special Issue entitled, “Focus on tumours in pet animals”, investigates our understanding of tumours in pet animals through fourteen publications; seven research papers, two reviews, and five case reports, all dedicated to broadening our understanding of cancer in pet dogs and cats. They include epidemiological studies, molecular profiling, investigations into novel potential therapeutic strategies, as well as assessment of the effectiveness of current treatment regimes, insights into prognostic biomarkers, detailed presentations of rare tumours and discussion of the translational role that tumours in pets can play in understanding cancer in humans.

There are two epidemiological studies in this Special Issue; one shines a spotlight on melanocytic tumours (MTs) in dogs and cats, and the other assesses the age at diagnosis of first malignancy in dogs and uses modelling to determine any influencing variables. MTs, which include melanocytoma (benign form) and melanoma (malignant counterpart), are relatively common in dogs and typically occur on the skin, digits, and oral cavities, with oral melanoma being one of most common malignant tumours in dogs. In contrast, MTs are less common in cats and most frequently occur in the eye, particularly the iris. Epidemiological studies have found that the prevalence, localisation, and age distribution of dogs and cats with MT can differ amongst countries, underscoring the importance of obtaining data from different countries. Lo Giudice and colleagues performed a retrospective study assessing canine and feline MT cases (n = 845 and n = 60, respectively) submitted to the Veterinary Pathology Service at the University of Perugia over a 19-year period [1]. The results from this study increase the richness of our understanding of the epidemiology of MTs in dogs and cats. The second epidemiological study evaluated the age at diagnosis in dogs with cancer, with a view to aiding the development of guidelines for cancer screening in companion animals. More than 14,000 canine neoplastic cases were coded according to the Vet-ICD-O-canine-1 (a system for coding canine neoplasms based on the International Classification of Diseases for Oncology (ICD-O) for humans (ICD-O-3.2)) [2], and stratified based on key parameters, with an event history analysis (EHA) model used to assess the influence of these parameters on the time at diagnosis of first malignancy [3]. This study clearly demonstrated that a “one-size-fits-all” approach to cancer screening in dogs is not suitable and offers insights into key considerations that should be factored into cancer surveillance approaches in dogs.

There are four research articles that focus on the treatment of cancer in dogs; two assessing current treatment regimens and two using in vitro experiments to investigate potential novel therapeutic strategies. Garcia-de la Virgen and colleagues [4] assessed the impact of toceranib phosphate and carprofen on the survival and quality of life in dogs with inflammatory mammary carcinoma (IMC). IMC is an infrequent type of mammary carcinoma that exhibits aggressive behaviour resulting in median survival times of a few months. Canine IMC shows strong expression of cyclooxygenase-2 and vascular endothelial growth factors (as well as other tyrosine kinase receptors), suggesting non-steroidal anti-inflammatory drugs (such as carprofen) and a multi-targeted tyrosine kinase inhibitor (such as toceranib phosphate) may prove useful in this setting. Although the study observed no responses (complete or partial), the treatment was well-tolerated, and the majority of dogs showed a clinical benefit through disease stabilisation and improved quality of life, suggesting this protocol may offer a valuable palliative option for dogs with IMC [4]. Dittmer and colleagues assessed the impact of multi-modal blockade of the renin–angiotensin system (RAS) inhibition in dogs during cancer treatment [5]. Although the traditional role of the RAS is the control of blood pressure/volume, more recently it has also been found to play a role in the tumour microenvironment, and, as such, numerous studies have investigated inhibiting different parts of the RAS as a therapeutic approach to cancer. As the RAS has a high level of redundancy (e.g., angiotensin II can be produced via numerous different pathways), treatments blocking multiple parts of the RAS are used in human cancer therapy. Thus, Dittmer and colleagues investigated whether a similar treatment regime, i.e., simultaneously blocking multiple parts of the RAS pathway (‘multimodal blockade’), could also be safely used to treat dogs with cancer [5]. Their pilot safety evaluation study, involving five dogs, found multimodal blockade of the RAS caused only mild adverse effects in some animals and thus provides the groundwork for future larger studies to assess the use of multimodal RAS blockade for the treatment of dogs with cancer [5].

The need to develop more treatment options for pet dogs and cats with cancer is undeniably clear. In vitro experimentation can provide vital first steps towards highlighting potential new therapeutic avenues. In this Special Issue, two articles perform experiments in cell lines and organoids to investigate potential novel therapeutic strategies in prostate and mammary carcinoma, respectively. Prostate cancer in canines has a poor prognosis due to its aggressive nature, metastatic propensity, and an absence of effective therapeutic protocols. As products derived from medicinal plants are increasingly playing an important role in cancer treatment (such as paclitaxel and vinblastine), Ramos de Moraes Calheiros and colleagues sought to test the anti-tumour effects of rich cannabidiol (CBD) and Δ9-tetrahydrocannabinol (THC) extract oils on canine prostate cancer cell lines [6]; the premise was that CBD and THC, the two main cannabinoids extracted from *Cannabis sativa*, have been shown to possess as antiproliferative and anti-invasive properties in different tumour types in humans. They found that CBD- or THC-rich extracts inhibited proliferation and induced cell death in two canine prostatic carcinoma cell lines [6], thus paving the way for further studies to investigate these compounds as potential therapeutic agents for prostate carcinoma in dogs. Mammary tumours are the second most frequent tumours seen in dogs (predominantly in older females), and ~50% are malignant at presentation. Surgery is the treatment of choice for canine mammary tumours (CMTs) as chemotherapy does not always offer a clear advantage; however, dogs with metastatic disease often have no successful treatment options. Therefore, more therapeutic options are needed, and in keeping with the success seen in human cancer patients, targeted therapies offer a promise of new hope. To this end, Ingelbert and colleagues [7] used two patient-derived organoid (PDO) lines, one from a CMT and one from non-neoplastic mammary tissue of the same dog, to perform a CRISPR screen to identify cancer-cell-specific vulnerabilities that could then represent potential therapeutic targets. Through two CRISPR/Cas9 dropout screens using libraries targeting epigenetic or druggable genes in the paired PDOs, they identified that the CMT cells showed a functional vulnerability for CDK2, that could be targeted with the existing inhibitor, PF3600 [7]. This study demonstrates the power of assessing genetic vulnerability using matched neoplastic versus non-neoplastic PDOs from a patient, to allow hundreds of genes to be functionally tested as potential therapeutic targets for that patient.

Molecular profiling of cancer is critical to identify potential molecular biomarkers (for diagnostic, prognostic, and/or therapeutic purposes). For example, numerous studies have reported the presence of the *BRAF^V595E^* mutation in the majority canine bladder urothelial carcinoma and prostate carcinoma samples, and there are now commercial tests available to detect the presence of *BRAF^V595E^* tumour cells in urine from dogs, as a screen for bladder or prostate cancer. However, not much is known about whether other tumour types in dogs also show the presence of this mutation. In this Special Issue, Bartel and colleagues [8] tested canine carcinoma samples (n = 227) from a large range of anatomical sites (n = 11), using two different antibodies that detected the mutant BRAF protein, and validated the findings using droplet digital PCR. BRAF^V595E^-mutated cases were found in prostate, bladder, and oral cavity samples, but no other sites. This study demonstrates that these antibodies are reliable diagnostic tools for detecting the BRAF^V595E^ mutation in formalin-fixed canine carcinoma samples, and that detection of this mutation in canine urothelial, prostatic, and oral squamous cell carcinomas provides a valuable opportunity to consider the use of BRAF inhibitors in dogs with these tumour types in the future.

There were two reviews in this Special Issue, both comparing tumours in pet dogs and cats to that seen in humans. Dogs and cats offer many advantages as a model for human cancers, including spontaneous tumour development, disease progression, tumour heterogeneity, genetic diversity among different breeds, exposure to similar environmental risk factors and similar co-morbidities (such as asthma, diabetes, and obesity). Importantly, it is essential to harness the power of ‘One Medicine’, such that what we learn in one species can, and should, be used to help the other. In the first review, Dell’Anno and colleagues [9] focussed on soft tissue sarcomas (STS), a diverse collection of tumour types arising from mesenchymal cells (including fibroblasts, adipocytes, and muscle). Current treatment approaches for STS in both dogs and humans typically involves surgery, radiotherapy, and/or chemotherapy; however, these tumours represent a clinical challenge due to their propensity for local recurrence and metastasis, and, as such, more treatment options are needed. The tumour microenvironment (TME) plays a crucial role in the tumourigenesis and progression of STS, such as through suppressing immune responses, modulating tumour behaviour, and promoting angiogenesis. Thus, understanding the complexities of the TME is critical if new tailored treatments are to be designed. To this end, Dell’Anno and colleagues [9] review the current understanding of STSs in canines and humans, emphasising the importance of the TME and exploring existing and new approaches for the treatment of STS, including immunotherapeutic strategies. In the second review, Cassali and colleagues [10] summarise decades of research from their group and that of their collaborators, together with other research in the literature, to provide a rich understanding of key factors in mammary pathology research. Encompassing canine, feline, and murine mammary tumours, this review addresses topics such as diagnostic, prognostic, and predictive factors; the TME (tumour stroma and tumour-associated inflammation); treatment options/approaches; and factors influencing tumour growth [10]. Cassali and colleagues conclude that each of these models have offered valuable contributions to veterinary and human oncology, playing pivotal roles in helping our understanding of tumour progression, therapeutic response, and treatment resistance in both humans and animals. Critically, there is no single “ideal experimental model”, and a detailed understanding of the strengths and limitations of the each model is necessary to maximise its scientific relevance [10].

Finally, this Special Issue is home to a fantastic collection of case reports: two related to rare tumour types, two related to novel diagnostic approaches, and one related to successful treatment of an aggressive tumour. Considering the rare tumour types, there is a report of a Yorkshire terrier with a post-mortem evaluation revealing a single large mass on a lobe of the lung, with numerous proliferative lesions of various sizes throughout all the lobes, and a solitary skin mass on the mid-thoracic body wall [11]. Histopathological and electron microscopy examination of the lung and skin masses led to a diagnosis of pulmonary adenocarcinoma with cutaneous metastasis [11]. Primary lung cancer is rare in dogs, and this is the first report of a canine with primary lung cancer that metastasized to the skin. In another report, an exploratory laparotomy led to identification of a mass in the jejunal wall of a Beagle dog with a history of recurrent ingestion of cotton-based toy fragments [12]. The mass was excised, and histopathological examination led to a diagnosis of osteoblastic osteosarcoma with fragments of cotton fibre material noted. Seven months post-surgery, liver metastases were identified, and the dog died three months later [12]. This case represents the first report of metastatic intestinal osteosarcoma that was potentially caused by frequent ingestion of textile fibres.

Considering novel diagnostic approaches, one report described the benefits of using an intraoperative frozen-section biopsy for the auxiliary diagnosis of transmural intestinal intermediate T-cell lymphoma in a Golden Retriever [13]. The authors wanted to demonstrate the diagnostic benefits of performing a frozen-section biopsy during surgery, by facilitating early diagnosis, accelerating therapeutic decision-making, and allowing for a clinical approach tailored to the individual animal’s needs [13]. In another report, the use of dynamic contrast-enhanced magnetic resonance imaging (DCE-MRI) was assessed for its clinical benefits in dogs with masses of epithelial and mesenchymal origin, given its routine use in human medicine [14]. More specifically, a preliminary case series of four dogs with benign or malignant tumours of epithelial or mesenchymal origin were assessed using DCE-MRI, versus computed tomography or conventional magnetic resonance imaging. The findings suggested that K^trans^ (a quantitative DCE-MRI parameter that represents tissue perfusion/vascular permeability and is related to malignant potential of the tumour) could be used to provide information about lesion characteristics in dogs, as well as a biomarker for evaluating the malignancy of a mass [14].

Finally, in a case report of an Abyssinian cat with lameness for several months, radiographic examination of the hind limbs revealed hypertrophic osteopathy in several locations, and examination of the abdominal radiographs identified a mass on the left kidney as the primary disease; the kidney was removed, and the mass was diagnosed as a renal cell carcinoma (RCC) [15]. Two months post-surgery, the cat was started on a renal prescription diet, in consideration of the remaining right kidney, and a follow-up revealed that the periosteal hyperplasia in some of the bones had completely regressed, with a follow-up at 6 months post-surgery showing that although the remaining lesions had not decreased, there were still no signs of lameness. At 4.5 years post-surgery, the cat developed polyuria and polydipsia, with blood tests suggesting chronic kidney disease (CKD). The cat ultimately lived till 12.5 years post-surgery; thus, this is the first report of RCC associated with hypertrophic osteopathy in a cat, in which long-term survival was achieved through surgical treatment alone [15].

Taken together, this Special Issue offers a rich insight into our current understanding of the epidemiology, molecular profiling, biomarkers, clinical presentations, and treatment options (both current and potential strategies) for cancer in pet dogs and cats, as well as looking at the role these animals can play as models for cancer in humans, as part of the One Medicine concept.

## References

[1] Lo Giudice A., Porcellato I., Giglia G., Sforna M., Lepri E., Mandara M.T., Leonardi L., Mechelli L., Brachelente C. (2024). Exploring the Epidemiology of Melanocytic Tumors in Canine and Feline Populations: A Comprehensive Analysis of Diagnostic Records from a Single Pathology Institution in Italy. Vet. Sci..

[2] Pinello K., Baldassarre V., Steiger K., Paciello O., Pires I., Laufer-Amorim R., Oevermann A., Niza-Ribeiro J., Aresu L., Rous B. (2022). Vet-ICD-O-Canine-1, a System for Coding Canine Neoplasms Based on the Human ICD-O-3.2. Cancers.

[3] Fonti N., Parisi F., Lachi A., Dhein E.S., Guscetti F., Poli A., Millanta F. (2024). Age at Tumor Diagnosis in 14,636 Canine Cases from the Pathology-Based UNIPI Animal Cancer Registry, Italy: One Size Doesn’t Fit All. Vet. Sci..

[4] Garcia-de la Virgen M., Del Portillo Miguel I., Maiques E., Pérez Roger I., Poch E., Borrego J. (2024). Impact of Toceranib Phosphate and Carprofen on Survival and Quality of Life in Dogs with Inflammatory Mammary Carcinomas. Vet. Sci..

[5] Dittmer K.E., Wetzel S., Odom T., Munday J.S., Flatt E.A., Wilson I.J., Hughes C., Tan S.T. (2024). Multimodal Blockade of the Renin–Angiotensin System in the Treatment of Cancer in Dogs Has Mild Adverse Effects in Some Dogs. Vet. Sci..

[6] Calheiros L.G.R.d.M., Pedro G., Oliveira da Silva T., Amorim R.M., Alves C.E.F., Laufer-Amorim R. (2024). In Vitro Antitumor Effect of Oils Rich in CBD and THC Cannabis Extract in Canine Prostate Carcinoma Cell Lines. Vet. Sci..

[7] Inglebert M., Dettwiler M., He C., Markkanen E., Opitz L., Naguleswaran A., Rottenberg S. (2025). Individualized Pooled CRISPR/Cas9 Screenings Identify CDK2 as a Druggable Vulnerability in a Canine Mammary Carcinoma Patient. Vet. Sci..

[8] Bartel A., Aupperle-Lellbach H., Kehl A., Weidle S., Aeschlimann L., Klopfleisch R., Brot S.d. (2024). Expression of Mutated BRAFV595E Kinase in Canine Carcinomas—An Immunohistochemical Study. Vet. Sci..

[9] Dell’Anno F., Giugliano R., Listorti V., Razzuoli E. (2024). A Review on Canine and Human Soft Tissue Sarcomas: New Insights on Prognosis Factors and Treatment Measures. Vet. Sci..

[10] Cassali G.D., Nakagaki K.Y.R., Salvi M., dos Reys M.P., Rocha M.A.N., de Campos C.B., Ferreira E., Rodrigues A.C.B., dos Reis D.C., Damasceno K.A. (2025). Canine, Feline, and Murine Mammary Tumors as a Model for Translational Research in Breast Cancer. Vet. Sci..

[11] Greyling A., van der Weyden L., Lensink A.V., O’Dell N. (2024). Pulmonary Adenocarcinoma with Cutaneous Metastasis in a Dog. Vet. Sci..

[12] Negoescu A., Gal C., Mihaila A., Mihaila C., Cătoi C., Taulescu M. (2024). Intestinal Osteosarcoma with Liver Metastasis in a Dog with a History of Recurrent Cotton-Based Toy Fragment Ingestion. Vet. Sci..

[13] Gaia de Sousa F., Milioli G., Neto J.A., Felice F.d., Chaves G., Pereira M., Lopes H., Wronski J., Nakagaki K., Beier S. (2025). Intraoperative Frozen Section Biopsy for the Auxiliary Diagnosis of Transmural Intestinal Intermediate T-Cell Canine Lymphoma. Vet. Sci..

[14] Cho C.-H., Kim J., Eom K. (2024). The Clinical Application of Dynamic Contrast-Enhanced MRI in Canine Masses of Mesenchymal and Epithelial Origin: A Preliminary Case Series. Vet. Sci..

[15] Tanaka T., Tanaka M., Tezuka T., Shimada K., Tanaka R. (2024). Successful Treatment of Renal Cell Carcinoma Associated with Hypertrophic Osteopathy in a Cat. Vet. Sci..

